# Aesthetic outcomes of inframammary fold recreation in two-stage, implant-based, breast reconstruction

**DOI:** 10.1186/s40064-016-3331-x

**Published:** 2016-09-26

**Authors:** Koichi Tomita, Kenji Yano, Akimitsu Nishibayashi, Shien Seike, Ko Hosokawa

**Affiliations:** Department of Plastic Surgery and Reconstructive Surgery, Osaka University Graduate School of Medicine, 2-2 C11 Yamadaoka, Suita, Osaka 565-0871 Japan

**Keywords:** Implant, Breast reconstruction, Inframammary fold, Aesthetic outcome

## Abstract

**Background:**

When the inframammary fold (IMF) is excised in mastectomy procedures for oncologic reasons, it must be recreated to restore a natural breast shape. Despite refinements in surgical techniques, postoperative loss of a well-defined IMF can occur. This study aimed to assess the outcomes of IMF recreation after two-stage, implant-based breast reconstruction.

**Methods:**

We retrospectively reviewed 75 consecutive patients who underwent unilateral, two-stage, implant-based breast reconstruction between 2013 and 2015 at the authors’ institution. Among them, IMF recreation was performed in 37 patients through a modified Nava’s internal method. Aesthetic outcomes of the recreated IMFs were evaluated by observer assessment of two criteria, and critical factors affecting IMF outcomes were also analyzed.

**Results:**

We found that contralateral breast ptosis (p < 0.05) and lack of postmastectomy radiotherapy (PMRT, p < 0.01) were significant predictors of better IMF outcomes. Nipple-sparing mastectomy and skin-sparing mastectomy resulted in better IMF outcomes, as compared with non-skin-sparing mastectomy (p < 0.05 for each), while no significant difference was observed between them in patients who did not undergo PMRT (p = 0.19). Similarly, larger implant volume, but not projection of implant, was a predictor of better IMF outcomes when limited to patients who did not undergo PMRT (p < 0.05). Age, body mass index, timing of reconstruction, and extent of overexpansion had no significant effect on IMF outcomes.

**Conclusions:**

Based on these critical factors, the shape of the reconstructed breast and the need for reshaping the contralateral breast can be predicted. Special attention should be paid to patients with non-skin-sparing mastectomy and PMRT. When these patients desire a medium- to large-sized ptotic breast, conversion to autologous reconstruction can achieve symmetrical breast reconstruction.

## Background

The inframammary fold (IMF) greatly affects the aesthetic outcomes of the reconstructed breast (Maclin et al. 2015). Since the IMF is defined by a unique dermal structure which is held in place by the superficial fascial system (Boutros et al. 1998; Riggio et al. 2000), it has to be recreated when excised in mastectomy procedures for oncologic reasons. As compared with breast reconstruction using autologous tissue, it is often difficult to create a well-defined IMF in implant-based breast reconstruction (Handel and Jensen 1992). To solve this problem, a number of methods have been reported, including both external (Pennisi 1977; Ryan 1982) and internal methods (Bogetti et al. 2007; Handel and Jensen 1992; Nava et al. 1998; Versaci 1987). In our institution, use of Nava’s internal method modified by Bogetti (Bogetti et al. 2007; Nava et al. 1998) is preferred due to its ability to achieve a well-defined, smooth IMF without additional scarring not only in breasts of various sizes, but also in lightly pendulous breasts. In our experience, aesthetic outcomes of the created IMF vary greatly on a case-by-case basis, prompting us to investigate factors that influence the outcomes of the created IMF. It would help not only in predicting the need for a contralateral side operation preoperatively, but also in suggesting conversion to autologous reconstruction when unsatisfactory outcomes are expected.

In this study, we retrospectively reviewed 37 consecutive patients who had undergone unilateral, two-stage, implant-based breast reconstruction, and who were operated upon by a single surgeon using a modification of Nava’s internal method. Aesthetic outcomes of IMF were evaluated by observer assessment of two criteria, and factors affecting outcomes were analyzed.

## Methods

### Patient selection and surgical procedure

This study was approved by the Ethics Committee of Osaka University (approval number 15520), and informed written consent to publish personal and medical information and all images was obtained from all patients. We retrospectively reviewed 75 consecutive patients who underwent unilateral, two-stage, implant-based breast reconstruction at Osaka University Medical Hospital between July 2013 and June 2015. Included were 37 patients (49 %) in whom the contralateral breast had a clear IMF over the entire length, as well as gland ptosis. Mean patient age was 51.5 years (range 30–74 years). According to the subjective ptosis scale, defined by Regnault (1976) 28 patients had no ptosis (but with a lightly pendant breast), eight patients had minor ptosis, and one patient had moderate ptosis. Immediate (23 cases) or delayed (14 cases) reconstruction was performed after nipple-sparing mastectomy (NSM, seven cases), skin-sparing mastectomy (SSM, 16 cases), or non-skin-sparing mastectomy (NSSM, 14 cases). In the first stage, a tissue expander (TE, Natrelle^®^133, Allergan Inc., Irvine, CA, USA) was placed in the subpectoral–subfascial plane (immediate reconstruction) or in the subpectoral-subcutaneous plane (delayed reconstruction). The extent of tissue expansion was determined by the quality of skin. In most cases, 0–20 % overexpansion was performed, and in selected cases (e.g., NSSM and radiotherapy cases), up to 50 % overexpansion was performed. In the second stage (usually 6–8 months after TE placement), the TE was replaced with a silicone implant (Natrelle^®^410, Allergan Inc., Irvine, CA, USA). Among 37 cases, postmastectomy radiotherapy (PMRT) was performed in 12 cases. In five cases (immediate reconstruction), PMRT was performed after TE placement with the TE in place, and in seven cases (delayed reconstruction) it was performed before TE placement. For patients who underwent immediate reconstruction and required PMRT, the second surgery was performed 6–8 months after finishing irradiation.

Exchange for the implant was performed using previous incisions. In the case of NSM, creation of the IMF was performed through a periareolar incision. After cutting capsule and soft tissue, including the superficial fascia, the IMF was created using a modified Nava’s internal method, as described by Bogetti et al. (2007). Instead of No.0 absorbable polyfilament sutures, we used 6–10 interrupted No.0 monofilament sutures (PDS^®^ Plus, Ethicon, USA), in expectation of long-lasting effects. All reconstruction procedures were performed by the first author (K.T.).

### Assessment of IMF score

Six months following exchange for the implant, standardized photographs were taken with patients standing straight and placing their hands on their iliac crests to allow for objective photographic assessment. Frontal photographs were used to evaluate IMF aesthetic outcomes. Two criteria were assessed on a three- or two-point scale by a blinded observer (a nurse). Criteria included the clarity of line and gland (implant) ptosis of the reconstructed breast (Table 1). Finally, a linear analogue scale from zero to three was calculated as a sum of the scores of these two criteria. A score of three was considered “excellent”, two as “good”, one as “fair”, and zero as “poor” (Table 1). Factors associated with patient characteristics [age, body mass index (BMI), breast ptosis, and postmastectomy radiation history], those associated with surgery (type of breast surgery, timing of reconstruction, and extent of overexpansion), and those associated with implant (implant volume and projection) were assessed as they relate to aesthetic outcomes of IMF.

**Table 1:** Inframammary fold score

Definition of IMF	2 (clear), 1 (unclear), or 0 (no line)
Gland (implant) ptosis	1 (yes) or 0 (no)
Overall: 3 (excellent), 2 (good), 1 (fair), 0 (poor)	

*IMF* inframammary fold

Statistical analysis was performed using statistical software (Statcel version 3). Data were analyzed using the Mann–Whitney *U* test, Kruskal–Wallis test, Spearman’s rank correlation coefficient, and Steel–Dwass test, as indicated. p < 0.05 was considered statistically significant.

## Results

Of the 37 included patients, 14 (37.8 %) were ranked as excellent, 9 (24.3 %) as good, 9 (24.3 %) as fair, and 5 (14.7 %) as poor. None of the patients experienced TE or implant failure by infection or extrusion. Some minor complications were observed, including minor mastectomy flap necrosis in three patients, minor infection of the TE in one patient, and hematoma in one patient. In all cases, good healing was achieved through conservative therapy.

Table 2 shows IMF aesthetic outcomes by patient and surgical factors. Patients who underwent PMRT showed significantly poorer IMF aesthetic outcomes than those who had no PMRT (p = 0.001). Preoperative breast ptosis positively affected IMF outcomes (p = 0.02). The type of breast surgery also significantly affected IMF aesthetic outcomes (p = 0.008). A Steel–Dwass test for multiple comparisons showed that NSM and SSM resulted in significantly better IMF outcomes than NSSM (p < 0.05 for each), whereas there was no significant difference in IMF outcomes between NSM and SSM. The type of breast surgery did not significantly affect IMF aesthetic outcomes when limited to patients without a history of PMRT (p = 0.19). Immediate reconstruction tended to show better IMF aesthetic outcomes than delayed reconstruction, but this difference was not statistically significant (p = 0.23). Other factors such as age, BMI, or extent of overexpansion did not affect IMF aesthetic outcomes.

**Table 2:** Patient and surgical factors affecting inframammary fold score

	Excellent	Good	Fair	Poor	*p*
Age					
<50 years	7 (41.2 %)	3 (17.6 %)	4 (23.5 %)	3 (17.6 %)	0.91^a^
≥50 years	7 (35.0 %)	6 (30.0 %)	5 (25.0 %)	2 (10.0 %)	
Body mass index (BMI, kg/m^2^)					
BMI < 22	6 (30.0 %)	8 (40.0 %)	5 (25.0 %)	1 (5.0 %)	0.96^a^
BMI ≥ 22	8 (50.0 %)	1 (6.3 %)	4 (25.0 %)	3 (18.7 %)	
Breast ptosis					
Grade 1 (no ptosis)	9 (33.3 %)	8 (30.0 %)	6 (22.2 %)	4 (14.8 %)	*0.02* ^**†**^
Grade 2 (minor ptosis)	4 (50.0 %)	1 (12.5 %)	2 (25.0 %)	1 (12.5 %)	
Grade 3 (moderate ptosis)	1 (100.0 %)	0	0	0	
Timing of reconstruction					
Immediate	10 (43.5 %)	6 (26.1 %)	5 (21.7 %)	2 (8.7 %)	0.23^a^
Delayed	4 (28.6 %)	3 (21.4 %)	4 (28.6 %)	3 (21.4 %)	
Postmastectomy radiotherapy					
No	13 (52.0 %)	6 (24.0 %)	6 (24.0 %)	0	*0.001* ^a^
Yes	1 (8.3 %)	3 (25.0 %)	3 (25.0 %)	5 (41.7 %)	
Type of breast surgery					
Nipple-sparing mastectomy	5 (71.4 %)	2 (28.6 %)	0	0	*0.008* ^‡^
Skin-sparing mastectomy	6 (37.5 %)	6 (37.5 %)	4 (25.0 %)	0	
Non-skin-sparing mastectomy	3 (21.4 %)	1 (7.1 %)	5 (35.7 %)	5 (35.7 %)	
Type of breast surgery^b^					
Nipple-sparing mastectomy	4 (100 %)	0	0	0	0.19^‡^
Skin-sparing mastectomy	6 (42.9 %)	5 (35.7 %)	3 (21.4 %)	0	
Non-skin-sparing mastectomy	3 (42.9 %)	1 (14.3 %)	3 (42.9 %)	0	
Overexpansion of TE (%)					
<10	9 (42.9 %)	4 (19.0 %)	6 (28.6 %)	2 (9.5 %)	0.59^a^
≥10	5 (31.3 %)	5 (31.3 %)	3 (18.8 %)	3 (18.8 %)	

*p* < 0.05 was considered statistically significant

*TE* tissue expander

^**†**^Spearman rank correlation coefficient for breast ptosis versus inframammary fold score

^‡^Kruskal–Wallis for type of breast surgery versus inframammary fold score

^a^Mann–Whitney U test for age, body mass index, timing of reconstruction, and radiation history versus inframammary fold score

^b^Results for patients without a history of radiotherapy

Table 3 summarizes IMF aesthetic outcomes by implant characteristics. Implants with higher volumes tended to produce better IMF aesthetic outcomes than implants with lower volumes (p = 0.18). When limited to patients without a history of PMRT, higher implant volume was a positive predictor of IMF outcomes (p = 0.04). Implant projection showed no significant effect on IMF aesthetic outcomes (p = 0.36).

**Table 3:** Implant characteristics affecting inframammary fold score

	Excellent	Good	Fair	Poor	*p*
Implant volume (mL)					
<200	0	2 (40.0 %)	3 (60.0 %)	0	0.18^†^
<300	3 (21.4 %)	6 (42.9 %)	3 (21.4 %)	2 (14.3 %)	
<400	9 (90.0 %)	1 (10.0 %)	0	0	
≥400	2 (25.0 %)	1 (12.5 %)	2 (25.0 %)	3 (37.5 %)	
Implant volume (mL)^a^					
<200	0	1 (25.0 %)	3 (75.0 %)	0	*0.04* ^†^
<300	3 (37.5 %)	4 (50.0 %)	1 (12.5 %)	0	
<400	8 (88.9 %)	1 (11.1 %)	0	0	
≥400	2 (50.0 %)	0	2 (25.0 %)	0	
Implant projection					
Low	1 (20.0 %)	0	3 (60.0 %)	1 (20.0 %)	0.36^†^
Moderate	7 (58.3 %)	3 (25.0 %)	1 (8.3 %)	1 (8.3 %)	
Full	4 (33.3 %)	4 (33.3 %)	3 (25.0 %)	1 (8.3 %)	
Extra full	2 (25.0 %)	2 (25.0 %)	2 (25.0 %)	2 (25.0 %)	

*p* < 0.05 was considered statistically significant

^†^Spearman rank correlation coefficient for volume and projection of implant versus inframammary fold score

^a^Results for patients without a history of radiotherapy

## Discussion

The IMF is not only a landmark of the female breast, but also an essential supporting structure which defines the shape of the whole breast. When the IMF is excised during mastectomy for oncologic reasons, the reconstructed breast loses its support, resulting in an unsatisfactory shape unless the IMF is recreated.

A number of methods of IMF recreation have been previously described. External methods, originally introduced by Pennisi and Ryan, can be used to create a well-defined IMF and augment the volume of the lower pole (Pennisi 1977; Ryan 1982). However, disadvantages, such as the presence of an additional long scar, and the risk of chronic irritation when wearing a brassiere (Versaci 1987), make this method less likely to be the first choice for implant-based breast reconstruction. The internal methods introduced by Versaci can overcome these drawbacks through the use of TEs and IMF reconstruction via an internal approach at the time of TE removal (Versaci 1987). Although the bulkiness and scalloped appearance of the IMF region was a drawback of the original method, modifications using the superficial fascia have overcome this (Bogetti et al. 2007; Handel and Jensen 1992; Nava et al. 1998). In creation of the new IMF, most authors used multiple interrupted sutures except for Nava et al. (1998) who described the use of one or two running sutures to consistently balance the tension of the suture line and to avoid the scalloped effect of multiple stitches.

Even with these technical improvements, internal methods still occasionally suffer from late loss of IMF definition. In the current study, we reviewed consecutive patients who underwent unilateral, two-stage, implant-based breast reconstruction using a modified Nava’s internal method, and investigated whether any factors served as outcome predictors.

**Fig. 1:**
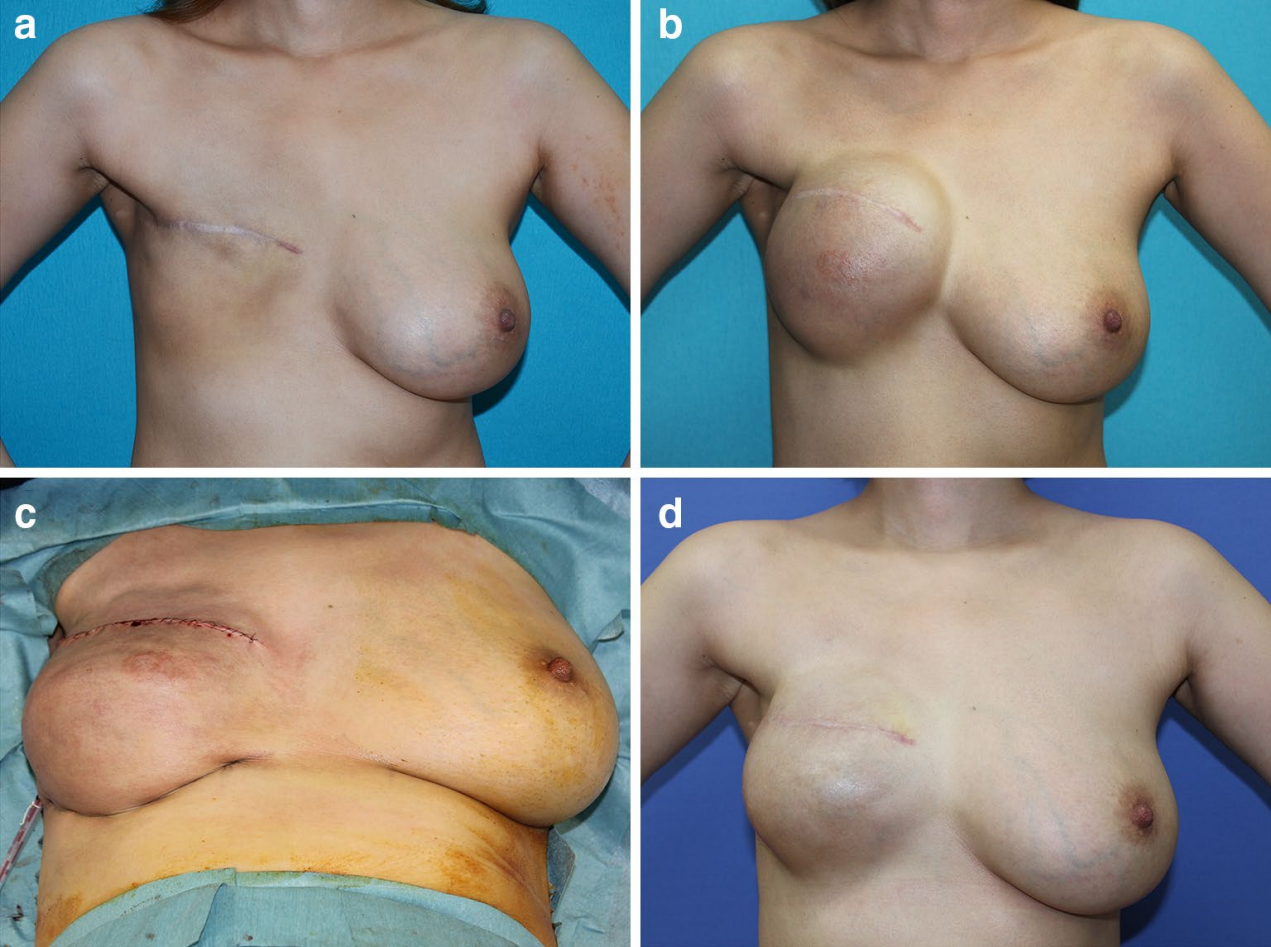
A 30-year-old patient who underwent delayed, right breast reconstruction following non-skin-sparing mastectomy and postmastectomy radiotherapy. **a** Preoperative view, **b** a tissue expander was inflated to 600 mL, **c** immediately postoperative view after exchange of the tissue expander for a 410 mL implant, **d** postoperative view. Note the loss of the defined inframammary fold. Future reduction of the contralateral breast is planned

**Fig. 2:**
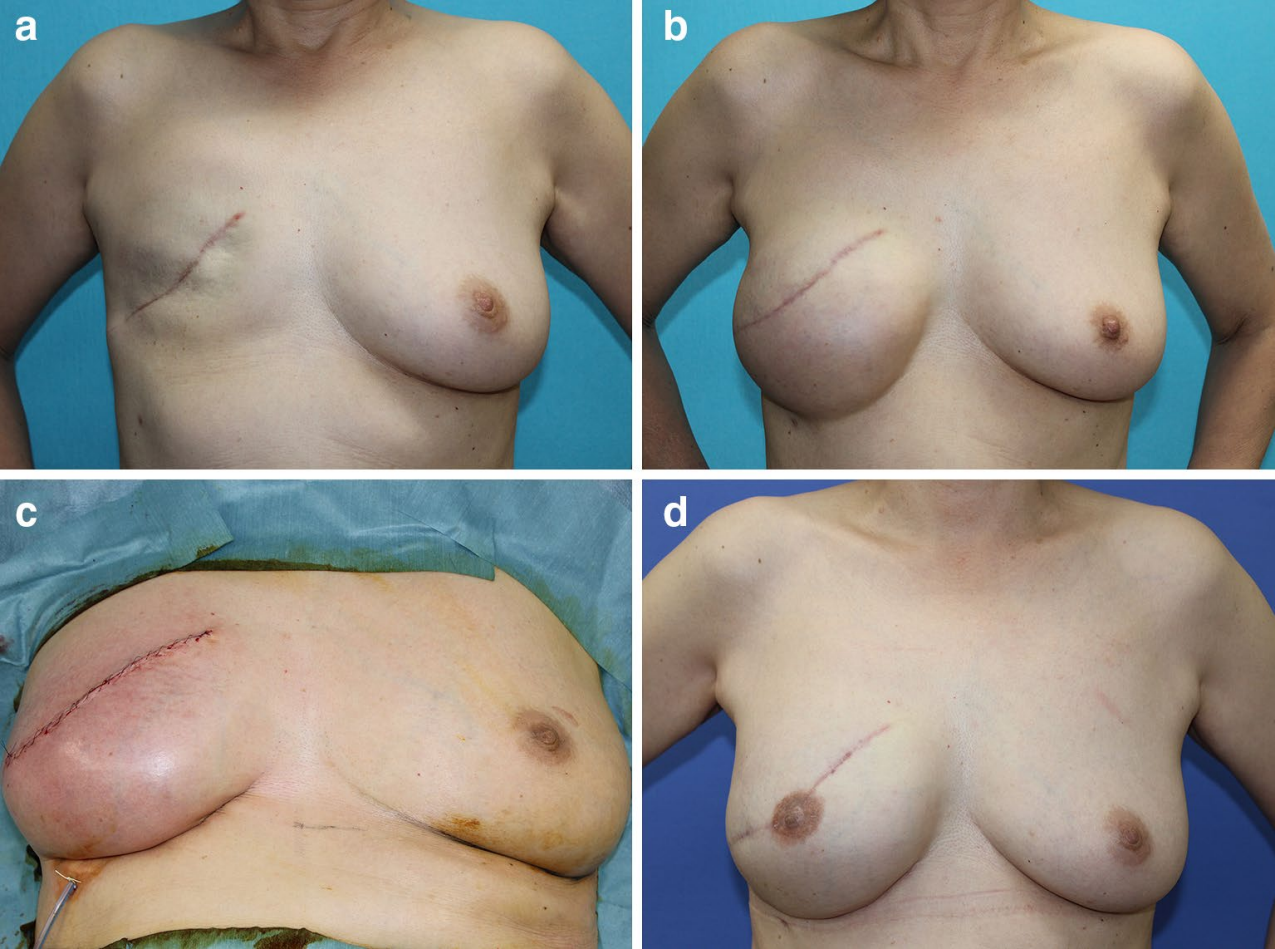
A 61-year-old patient who underwent delayed, right breast reconstruction following non-skin-sparing mastectomy. **a** Preoperative view, **b** a tissue expander was inflated to 475 mL, **c** immediate postoperative view after exchange for a 470 mL implant, **d** postoperative view. A well-defined inframammary fold and a natural breast shape were reconstructed

Since skin expansion with TEs is a fundamental part of the internal method we used, we suspected that factors related to the amount and elasticity of breast skin might affect the IMF outcome. As expected, PMRT, which induces subcutaneous fibrosis and decreased elasticity (Kim et al. 2013), was predictive of negative IMF outcomes. Similarly, NSM and SSM, in which most of the original breast skin is preserved, were advantageous compared to NSSM, in which a certain amount of breast skin is excised. It is notable that, of the seven patients who underwent both NSSM and PMRT, five and two patients were scored as “poor” and “fair,” respectively. Although good breast shape was obtained immediately after surgery in these patients, natural breast shape was lost during the postoperative process (Fig. 1). When limited to patients without PMRT, the type of breast surgery was not a significant predictor of IMF outcomes. In such a situation, a pendulous breast with a well-defined IMF could be reconstructed via adequate skin expansion even after NSSM (Fig. 2). Regarding skin expansion, overexpansion has been reported as a useful technique for reconstructing large and protuberant breasts (Versaci 1987), but it did not positively affect IMF outcomes in the present study.

The timing of reconstruction was not a significant predictor of IMF outcomes. Since both immediate and delayed reconstruction have potential advantages and disadvantages, they may counterbalance each other. For instance, immediate reconstruction is advantageous in that skin expansion is initiated before the completion of subcutaneous scar formation. This technique also has the disadvantage that TE placement in the subpectoral–subfascial plane is advised in order to reduce postoperative complications, resulting in the disturbance of skin expansion in the lower pole by myofascial constriction (Nava et al. 1998). Conversely, in delayed reconstruction, TEs can be safely placed in the subpectoral-subcutaneous plane, thus facilitating expansion in the lower pole, while subcutaneous scar formation is usually completed before the initiation of skin expansion. Additional data using the same subpectoral pocket might be helpful to further assess the significance of this factor.

We also paid attention to the characteristics of implants. When limited to patients without PMRT, larger implant volume was a significant predictor of better IMF outcomes. Projection of the implant, however, was not found to be a significant predictor of IMF outcomes. This may be related to the fact that increased breast projection with implants has some corrective effects on breast ptosis (Parsa and Parsa 2006), while implant volume was frequently medium to large in patients in need of high-projection implants in our study. Further study is required to understand the effects of implant volume and projection on recreated IMF definition.

## Conclusions

To the best of our knowledge, predictors of IMF outcomes after implant-based breast reconstruction have not been previously assessed. Contralateral breast ptosis, lack of PMRT, less invasive breast surgeries such as NSM and SSM, and larger implant volume (when limited to patients without PMRT) were identified as significant predictors of better IMF outcomes. These critical factors could be used to predict the shape of the reconstructed breast, as well as the need to reshape the contralateral breast. Close attention should be paid to patients with NSSM and PMRT. When these patients desire a medium- to large-sized, ptotic breast, conversion to autologous reconstruction is an option, since external methods, which fix the IMF more firmly, can cause complications such as chronic irritation or extrusion in such patients.

## Abbreviations

IMF: inframammary fold
NSM: nipple-sparing mastectomy
SSM: skin-sparing mastectomy
NSSM: non skin-sparing mastectomy
PMRT: postmastectomy radiotherapy
